# Round Goby (*Neogobius melanostomus*) impacts on benthic fish communities in two tributaries of the Great Lakes

**DOI:** 10.1007/s10530-022-02816-4

**Published:** 2022-05-27

**Authors:** Keith McAllister, D. Andrew R. Drake, Michael Power

**Affiliations:** 1 Department of Biology, University of Waterloo, 200 University Avenue West, Waterloo, ON N2L 3G1 Canada; 2 Great Lakes Laboratory for Fisheries and Aquatic Sciences, Fisheries and Oceans Canada, Burlington, ON L7S 1A1 Canada

**Keywords:** Round Goby, Native benthic fishes, Aquatic invasive species, Great Lakes tributaries

## Abstract

Numerous fish species in the Laurentian Great Lakes have been negatively impacted by the establishment of the invasive Round Goby (*Neogobius melanostomus*). However, limited understanding exists as to how Round Goby has impacted small-bodied native benthic fishes after its secondary invasion into tributaries of the Laurentian Great Lakes. To investigate Round Goby impacts on darter species (family Percidae) in tributary ecosystems, catch per unit area (CPUA) of native and non-native fishes from two riverine ecosystems in Southwestern Ontario (Ausable River, Big Otter Creek) were analyzed. Spatial analyses indicated Round Goby CPUA was highest proximate to the Great Lakes, with a sharp decline in CPUA at sites upstream from each lake (Round Goby CPUA approached zero after 18 and 14 km in the Ausable River and Big Otter Creek, respectively). There was some evidence of a negative relationship between the CPUA of Round Goby and several darter species along the tributary gradients, with moderately negative co-occurrence between Round Goby and Rainbow Darter in the Ausable River and Johnny Darter and Percidae species overall in Big Otter Creek. However, overwhelming evidence of negative associations between Round Goby and all darter species was not found. The negative relationship between the CPUA of Round Goby and some darter species was observed over similar time periods since establishment but greater spatial scales than in previous studies, and therefore has important implications for understanding the ecological impacts of Round Goby in tributary ecosystems.

## Introduction

Aquatic invasive species (AIS) can drastically affect the ecosystems they invade with many AIS having led to significant declines in native fishes (Chick et al. 2020; Cucherousset and Olden 2011; Fetterolf Jr 1980; Hermoso et al. 2011; Ogutu-Ohwayo 1990). Generally, ecological impacts increase as the density of the invader increases, with impacts to native species occurring through a variety of ecological mechanisms that include competition, predation, behavioural effects, and food web changes (Bradley et al. 2019; Gallardo et al. 2016). At high invader densities, increased intraspecific interactions may lead to diminished ecological impacts, as has been experimentally demonstrated for invasive Round Goby (*Neogobius melanostomus*) (Kornis et al. 2014). AIS are often more aggressive and grow larger than native species, which may prevent native species from accessing optimal habitat and dietary resources as a result of interference competition (Persson, 1985; Pimm et al. 1985; St-Pierre et al. 2006; Volpe et al. 2001). Exploitative competition for food resources may also simultaneously occur between trophically similar invasive and native species, resulting in lower growth of native species compared to allopatric conspecifics (Seiler and Keeley, 2009). Abundance declines in numerous native fish species have been linked to competitive interactions with AIS that have negatively affected population vital rates (e.g. growth and fecundity) (Cucherousset and Olden, 2011). AIS may also alter food web structure, often by increasing food chain length or modifying basal trophic levels (Cucherousset et al. 2012).

The Round Goby is native to the Ponto-Caspian region, but was transported to North America in the ballast water of commercial ships. It was first detected in the St. Clair River in 1990 (Jude et al. 1992) and subsequently spread to all five Great Lakes within five years (Corkum et al. 2004). Round Goby quickly attained high abundance in nearshore lake habitats and its occurrence has been linked to reduced abundance of several benthic fishes in the Great Lakes basin, including Mottled Sculpin (*Cottus bairdii*), Logperch (*Percina caprodes*), Johnny Darter (*Etheostoma nigrum*), Rainbow Darter (*Etheostoma caeruleum*), and Tessellated Darter (*Etheostoma olmstedi*) (Balshine et al. 2005; Bergstrom and Mensinger 2009; French and Jude 2001; Krakowiak and Pennuto 2008; Lauer et al. 2004; Morissette et al. 2018).

The initial establishment of Round Goby in the Great Lakes was followed by secondary expansion into numerous Great Lakes tributaries (e.g. Ontario: Big Otter Creek in 2002, Trent River in 2003, Thames River in 2003, Grand River in 2005, Ausable River in 2007) (Poos et al. 2010; Raab et al. 2018; Raby et al. 2010). Round Goby impacts on the native fish communities in North American rivers have varied despite the expectation that Round Goby would outcompete small native benthic fishes such as darters and other Percidae species (Jude et al. 1992; Poos et al. 2010). For example, Rainbow Darter and Johnny Darter were not detected in any of the sampled streams within studied New York state tributaries of Lake Erie after Round Goby establishment, despite the historical presence of the darter species (Krakowiak and Pennuto 2008). Conversely, an approximately 11-fold increase in Round Goby in numerous Lake Michigan tributaries had no detectable negative effects on the abundance of several native benthic species, including: Johnny Darter, Blackside Darter (*Percina maculata*), and Fantail Darter (*Etheostoma flabellare*) over a four-year study period, although the result may have been linked to the recency of the Round Goby invasion at the selected study sites (Kornis et al. 2013). Similarly, Round Goby had no apparent effect on Blackside Darter abundance in two Lake Michigan tributaries (Silver Creek and Pigeon River), which was also attributed to the recency of invasion (Malone 2016). Thus, Round Goby impacts appear to be context-dependent and may vary widely depending on ecosystem factors, including native community composition, food web dynamics, time since invasion, and Round Goby density.

The few studies that have examined Round Goby impacts in North American rivers have been limited with respect to their spatial breadth as studied sites have typically been located near invaded lacustrine environments, e.g. < 20 km (Kornis et al. 2013; Krakowiak and Pennuto 2008; Malone 2016). Conversely, several European studies have investigated Round Goby impacts in riverine environments at greater spatial scales (up to 250 km) and have observed differences in Round Goby impacts when compared to North American studies. In European rivers, Round Goby impacts vary spatially across longitudinal river gradients (Borcherding et al. 2011; Brandner et al. 2018; Cerwenka et al. 2018). For example, Round Goby has become the dominant fish species in terms of relative abundance in the upper Danube River (Cerwenka et al. 2018) and directly contributed to the declines of many specialized native species (Mueller et al. 2018). When comparing along the invasion gradient in the upper Danube, Round Goby populations located near the invasion front are composed of more females, larger and better-conditioned adults, and have a lower proportion of juveniles than in areas where Round Goby has been established for longer time periods (Brandner et al. 2018). Given these differences, it is important to further examine the variation in Round Goby impacts on fish communities in North American lotic environments at greater longitudinal scales.

In the Great Lakes basin, numerous Percidae species are facing population declines due to anthropogenic threats (e.g. pollution, excess nutrients, sedimentation, impoundment effects) and many are protected under Canadian federal and provincial conservation legislation (e.g. Eastern Sand Darter (*Ammocrypta pellucida*), River Darter (*Percina shumardi*), Channel Darter (*Percina copelandi*)) (Pratt et al. 2016). Round Goby expansion and establishment in the tributaries of the Great Lakes, therefore, is believed to pose further threats to these and other darter species due to probable competition for similar dietary and habitat resources, and via egg predation by Round Goby (French and Jude 2001; Raab et al. 2018). For example, Percidae species have displayed increased specialized feeding on Chironomidae in tributaries also occupied by Round Goby (Firth et al. 2020), likely because Round Goby deplete Ephemeroptera, Plecoptera, and Trichoptera (EPT) and other grazers and shredders that would otherwise constitute important food resources for native fish species (Krakowiak and Pennuto 2008; Pennuto et al. 2018). There is also evidence of Round Goby displacing darters to different microhabitats (Abbett et al. 2013; Reid 2019), with experimental studies indicating that Round Goby can outcompete Logperch for their preferred habitat (Balshine et al. 2005; Leino and Mensinger 2017). Collectively, previous studies have provided sound evidence that darter species are the most likely fishes to be impacted by the establishment of Round Goby in tributaries of the Great Lakes (Raab et al. 2018; Firth et al. 2020).

Past studies investigating Round Goby in small tributaries flowing directly into the Great Lakes have mostly focused on fish community impacts at limited spatial scales (i.e. downstream reaches < 20 km upstream from the river mouth). Analyzing the effects of Round Goby at broader spatial scales within invaded tributaries, however, is critical to develop a broader understanding as to how invaded riverine ecosystems will eventually be impacted. Thus, the main objective of this study is to evaluate whether the presence of Round Goby is associated with lower relative abundance of Percidae species (specifically darters and Logperch) and lower diversity, evenness, and species richness of the fish communities in invaded tributaries of the Great Lakes. Specifically, we hypothesize that: (1) sites with higher relative abundance of Round Goby will be associated with lower relative abundance of other benthic Percidae species (Greenside Darter (*Etheostoma blennioides*), Blackside Darter, Johnny Darter, Rainbow Darter, and Logperch) and (2) tributary reaches with high Round Goby abundance will exhibit lower diversity, evenness, and species richness.

## Methods

### Field sampling

Forty-five sites in the Ausable River (a tributary of Lake Huron, river mouth: 43°23′N, 81°91′W) and fifty sites in Big Otter Creek (a tributary of Lake Erie, river mouth: 42°64′N, 80°81′W) were sampled by Fisheries and Oceans Canada (DFO) field crews for this study (Barnucz et al. 2020). Sampling in the Ausable River was completed August 15th—September 28th, 2017 (36 sites) and July 24th—July 26th, 2018 (9 sites) using multiple gears: a siamese trawl (3.0 m tow ropes, two 6 kg 0.5 × 0.3 m otter doors, 3.0 mm mesh size, 2.4 m wide, 4.3 m length), a straight seine with chain (3.0 mm mesh, 6.0 m length), and a bag seine (3.0 mm mesh, 9.1 m length). The 9 sites from 2018 spanned the upstream-most (117–126 km upstream from the river mouth) sampled section in the Ausable River. Portions of the Ausable River consisted of habitats inaccessible by wading or dominated by large physical obstructions (e.g. woody debris). Thus, gear selection in the Ausable River was adapted to the site-specific conditions to optimize sampling efficiency. In Big Otter Creek, sampling occurred between July 9–19th and September 24–26th, 2018 using only a bag seine (3.0 mm bag mesh, 3.0 mm wing mesh, 9.1 m length). Seining in both tributaries was completed in a downstream direction at each site with three consecutive hauls. A time of roughly 5 min between hauls was designated to allow fish to repopulate the fished area. To minimize disturbance, survey crews began sampling in the downstream-most sampling unit and then worked upstream towards the next unit (allowing for the release of captured fishes downstream of the site to avoid recapture in sites upstream). Captured fishes were kept in bankside aquaria, identified to species, and enumerated for each haul. A subset of fishes was kept and preserved in a 10% formalin solution to confirm species identification and for future analyses. Additional sampling details can be found in Barnucz et al. (2020).

### Aquatic habitat sampling

Aquatic habitat variables were measured at the midpoint of each sampling site after fishes were collected. Water temperature (°C), conductivity (μS), turbidity (NTU), and dissolved oxygen (mg/L) were measured roughly 0.1 m below the water surface with a YSI EX02 Multiparameter Sonde (Xylem Inc., White Plains, NY). Substrate composition was determined by taking a grab sample of bed material to record percent composition of the sample based on median particle diameter (clay: 0–0.002 mm, silt: 0.002–2 mm, gravel: 2–40 mm, cobble: 40–256 mm, and boulder: > 256 mm). Channel depth was measured at three separate locations within the boundaries of the seined area (shallow, mid-depth, and deep) with a metre stick. Stream velocity (m/s) was similarly measured in three separate locations (slowest, mid-velocity, fastest) with a Swoffer 2100 current velocity meter (Swoffer Instruments, Sumner, WA) deployed at roughly 50% of the stream depth. Wetted stream channel width (m) was measured at the midpoint of the seining site (Ausable River) or river reach (Big Otter Creek) perpendicular to the bank with the use of a Nikon Laser 1200S waterproof laser range finder (Nikon Canada Inc., Mississauga, ON). Site latitude and longitude were recorded using a Garmin Montana 600 handheld GPS unit (Garmin Ltd., Olathe, KS).

### Statistical analysis

Catch per unit area sampled (CPUA) was determined as the aggregate number of captured fish × seined area (m^2^)^−1^. Broken-stick regression was used to compare CPUA of Round Goby and other Percidae species (Johnny Darter, Blackside Darter, Greenside Darter, Rainbow Darter, and Logperch in the Ausable River and Johnny Darter, Blackside Darter, and Logperch in Big Otter Creek) with site distance from the river mouth [measured along the river channel using the linear measuring tool from ArcGIS Online® software (Esri Canada, Toronto, ON, Canada)] as follows:

$$Y=a+{b}_{1} (X)+ {b}_{2 }(Z)(X-T)$$

where *a* is the intercept, *b*_1_ is the initial slope coefficient, *b*_2_ is the slope modifying coefficient, *T* is the breakpoint of the line where the slope changes from *b*_1_ to (*b*_1_ + *b*_2_), and *Z* defines where the breakpoint occurs ($Z=0$ if $X<T$ and $Z=1$ if $X>T$). The breakpoint was determined following methods for estimating piecewise regression models with unknown breakpoints using the ‘segmented’ package in R (Hudson 1966; Muggeo 2021). All statistical analyses were completed using R version 4.0.4 (R Core Team 2021).

Only species present at greater than 5% of sites were included in the statistical analyses as the focus of the study was on determining how Round Goby may be affecting the more common Percidae species along upstream/downstream tributary gradients. While Round Goby impacts may be most severe on rare Percidae species, it would be difficult to determine patterns in relative abundance when a species is detected in < 5% of sampled sites as rarity can obscure the ability to detect biologically significant differences between sites (Hawkins et al. 2000). Similarly, Kornis et al. (2013) restricted analyses to non-Round Goby species detected at > 5% of sites when testing associations between various species relative abundance and environmental data. Linear regressions were performed to test for the significance of correlations between the CPUA of Round Goby (excluding sites where Round Goby CPUA = 0) and the Percidae species in both tributaries. Additionally, Phi coefficients (Yule 1912) were calculated to test for relationships between the presence/absence of Round Goby and Percidae species as follows (Alofs and Jackson 2015; Jackson et al. 1989):

$$\varphi =\frac{ad-bc}{\sqrt{\left(a+b\right)\left(a+c\right)\left(c+d\right)(b+d)}}$$

where *a*, *b*, *c*, and *d* are the entries from two-by-two contingency tables which define the number of sites where Round Goby and the Percidae species are both absent, Round Goby is absent and the Percidae species are present, Round Goby is present and the Percidae species are absent, and Round Goby and the Percidae species are both present, respectively. Phi coefficients represent pairwise associations between species independent from relative abundance and range from negative one (perfect negative association) to one (perfect positive association).

### Fish community indices

To analyze whether the impact of Round Goby differed spatially within each tributary, sampling sites were grouped into lower, middle, and upper sites based on longitudinal distance from the river mouth. In Big Otter Creek, lower, middle, and upper sites were 13.1–14.1 (n = 13), 35.8–49.9 (n = 9), and 71.2–83.6 km (n = 5) upstream from Lake Erie, respectively. In the Ausable River, lower, middle, and upper sites were 11.2–17.7 (n = 16), 32.2–75.6 (n = 12), and 91.6–126.4 km (n = 17) upstream from Lake Huron, respectively. Within each grouping, sites in the middle of the designated section were selected for use in statistical comparisons to ensure sufficient distance between each site grouping. Distances between the site groupings thus exceeded the linear home range distance typically observed for small benthic fishes (Minns 1995; Woolnough et al. 2009). Sites in both tributaries were grouped into 15 km clusters with CPUA of Round Goby and Percidae species averaged among the clusters to test for spatial autocorrelation using Moran’s I from the package ‘ape’ (Paradis et al. 2022). Sites in close proximity to low-head dams or the Great Lakes were excluded as Round Goby is known to exist at high densities in these locations (Krakowiak and Pennuto 2008; Kornis et al. 2013; Malone 2016; Raab et al. 2018; May et al. 2020). Thus, in analyses comparing the three site groupings, 23 sites from Big Otter Creek were excluded and no sites in the Ausable River were excluded. Additionally, differences in mean habitat conditions of site groupings in each tributary were examined using one-way ANOVAs with post-hoc Tukey tests used to compare means between site groupings where necessary.

Diversity and evenness were used to characterize and compare the lower, middle, and upper river fish communities in both tributaries (Table 1), with species diversity determined using the Shannon–Wiener diversity index (Shannon and Weaver 1949; Spellerberg and Fedor 2003). Evenness was calculated using Smith and Wilson’s Index of Evenness as it is independent of species richness and sensitive to both rare and common species in the community (Smith and Wilson 1996). Several CPUA categories (CPUA of all fishes excluding Round Goby, CPUA of all fishes including Round Goby, and CPUA of Round Goby) were also compared between site groupings in each tributary. Additionally, the Morisita-Horn index (Horn 1966), modified from Morisita (1959) and noted as one of the more robust measures of overlap (Smith and Zaret 1982), was also calculated by adding CPUA of species in each site grouping to determine the similarity in species composition between site groupings.

**Table 1:** Metrics used to compare fish communities within site groupings in each tributary where *s* defines the number of species, *p*_*i*_ defines the proportion of individuals in the sample belonging to the *i*th species, *n*_*i*_ defines the number of individuals of species *i* in the sample, *n*_*j*_ defines the number of individuals of species *j* in sample, *x*_*i*_ is the number of times species *i* is represented in the total* X* from one sample, and *y*_*i*_ is the number of times species *i* is represented in the total *Y* from another sample

Metric	Formula
Shannon–Wiener index (*H*)	$H= -\sum_{i=1}^{s}{p}_{i}\;\mathrm{ln}{p}_{i}$
Smith and Wilson’s Index of Evenness (*E*_*var*_)	${E}_{var}=1-\left(\frac{2}{\pi }\right) \left[\mathrm{arctan}\left\{\frac{\sum_{i=1}^{s} {\left({log}_{e}({n}_{i}) - \sum_{j=1}^{s}{log}_{e}({n}_{j}) /s\right)}^{2}}{s}\right\}\right]$
Catch per unit area (*CPUA*)	$CPUA=\frac{\textit{Number of fish}}{\textit{Seined area} ({m}^{2})}$
Morisita-Horn Index of Similarity (*C*_*H*_)	${C}_{H}= \frac{2\sum_{i=1}^{s}{x}_{i}{y}_{i}}{\left(\frac{\sum_{i=1}^{s}{{x}_{i}}^{2}}{{X}^{2}}+\frac{\sum_{i=1}^{s}{{y}_{i}}^{2} }{{Y}^{2}}\right) XY}$

### Species accumulation curves

Species accumulation curves were generated to compare the accumulation of fish species with an increasing number of sites in each tributary. The method involved using an exact calculation for site-based species richness (Ugland et al. 2003; Colwell et al. 2004; Kindt et al. 2006), given by:

$$\widehat{{S_{n}}} =\mathop \sum \limits_{{i = 1}}^{S} \left( {1 - p_{i} } \right),\quad{\text{where}}\; p_{i} = \frac{{\left( {\begin{array}{c}{N - f_{i} }\\ n\\ \end{array} } \right)}}{{\left( {\begin{array}{c}N\\ n\\ \end{array} } \right)}},$$

and where *f*_*i*_ is the frequency of species *i*, *S* is the number of species, with the expected number of species in a community rarefied from *N* to *n* individuals. Species accumulation curves for each site grouping were created using randomization to compute the curves and corresponding 95% confidence intervals with the ‘vegan’ and ‘BiodiversityR’ packages (Oksanen 2020; Kindt 2021).

### Local richness estimators

To quantitatively estimate the theoretical upper limit of the number of species present for each site grouping, local richness estimates were calculated (Chao and Chiu 2016). For comparative purposes, the function ‘specpool’ from the package ‘vegan’ was used to generate estimates of local species using the non-parametric and abundance-based Chao1 (Oksanen 2020). The ‘specpool’ function assumes that the number of undetected species is related to the number of rare species (i.e., those only seen once or twice) (Oksanen 2020).

## Results

### Round Goby impacts on relative abundance of Percidae species

**Fig. 1:**
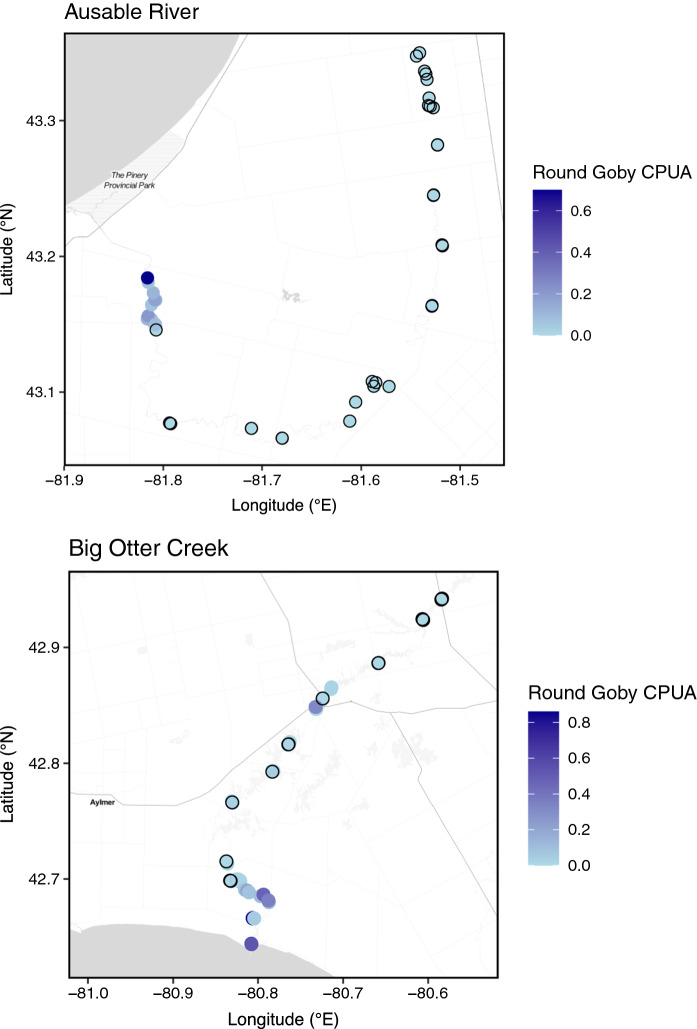
Round Goby CPUA (number of fish × (m^2^)^−1^) in sampling locations along the Ausable River in 2017 and 2018 (n = 45 sites) and Big Otter Creek in 2018 (n = 50 sites). Sites where Round Goby was not detected (CPUA = 0) are outlined in black

**Fig. 2:**
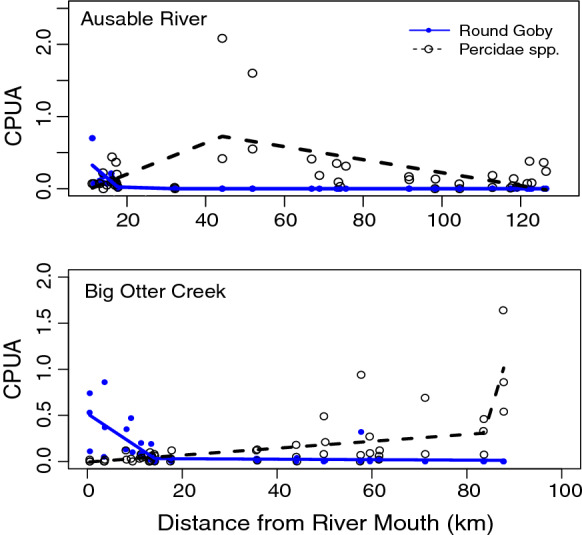
Broken-stick regression models and CPUA for Round Goby (solid blue line, blue dots; Adj. R^2^ = 0.51, 0.53 for the Ausable River and Big Otter Creek, respectively) and Percidae species (dashed black line, open dots; Adj. R^2^ = 0.26, 0.57 for the Ausable River and Big Otter Creek, respectively) at sampling locations along the Ausable River and Big Otter Creek in relation to the distance from river mouth (km). Percidae species from the Ausable River used in the model included: Johnny Darter (*Etheostoma nigrum*), Blackside Darter (*Percina maculata*), Greenside Darter (*Etheostoma blennioides*), Rainbow Darter (*Etheostoma caeruleum*), and Logperch (*Percina caprodes*), whereas Percidae species from Big Otter Creek used in the model included: Johnny Darter, Blackside Darter, and Logperch. Percidae species collected in < 5% of sites within a tributary were excluded from analyses

**Fig. 3:**
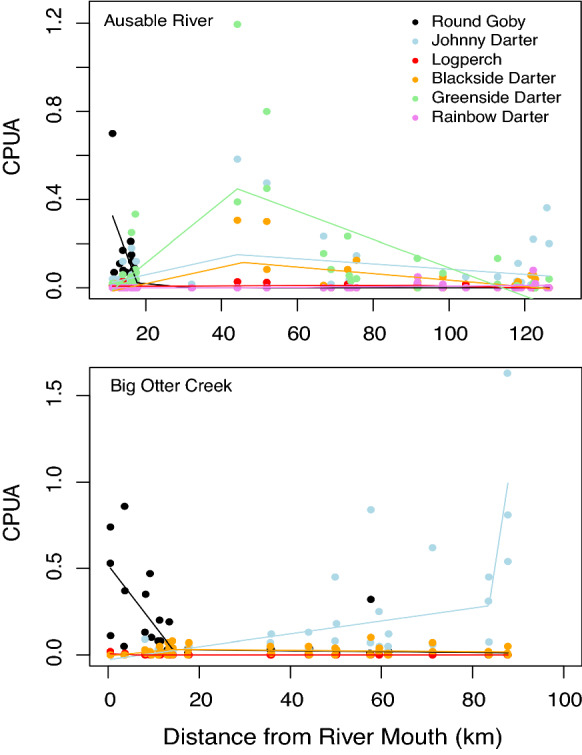
Broken-stick regression models and CPUA for Round Goby and individual Percidae species in sampling locations along the Ausable River (top panel) and Big Otter Creek (bottom panel) in relation to the distance from river mouth (km). Percidae species displayed for the Ausable River included: Johnny Darter (*Etheostoma nigrum*), Blackside Darter (*Percina maculata*), Greenside Darter (*Etheostoma blennioides*), Rainbow Darter (*Etheostoma caeruleum*), and Logperch (*Percina caprodes*), whereas Percidae species displayed for Big Otter Creek included: Johnny Darter, Blackside Darter, and Logperch. Percidae species collected in < 5% of sites within a tributary were excluded from analyses

In both rivers, Round Goby CPUA declined sharply with upstream distance from the tributary mouth (Figs. 1 and 2) whereas the CPUA of the Percidae species increased. In the Ausable River, an overall increasing then decreasing trend in CPUA of Percidae species was largely driven by Greenside Darter, Blackside Darter, and Johnny Darter in sites between 44 and 52 km upstream from Lake Huron (Fig. 3). Logperch and Rainbow Darter CPUA remained consistently low as distance from the mouth of the Ausable River increased (Fig. 3). The overall increasing trend in the relative abundance of Percidae species in Big Otter Creek as a function of distance from the river mouth was largely driven by Johnny Darter while the CPUA of the other species remained consistently low (Fig. 3). In Big Otter Creek, an anomaly from the low occurrence of Round Goby at distances greater than 14 km from the river mouth occurred 58 km upstream where 32 Round Goby were caught (Figs. 1 and 2). Round Goby were not detected after 18 and 62 km upstream, respectively, in the Ausable River or Big Otter Creek.

In the Ausable River, CPUA of Round Goby was not significantly correlated with CPUA of Blackside Darter, Greenside Darter, Logperch, or Johnny Darter (Table 2). Similarly, no significant associations between Round Goby CPUA and other Percidae species were noted in Big Otter Creek. Phi coefficients showed a similar lack of strong association between Round Goby and other studied fish species. In the Ausable River, Round Goby showed a moderately positive association with Logperch (0.26), Johnny Darter (0.21), and Greenside Darter (0.3) and a moderately negative association with Rainbow Darter (− 0.25). Blackside Darter (− 0.06) and Percidae species overall (0.06) showed no meaningful associations with Round Goby. In Big Otter Creek, there were weak associations with Logperch (0.18) and Blackside Darter (− 0.16), and moderately negative associations with Johnny Darter (− 0.31) and Percidae species overall (− 0.28).

**Table 2:** Summary of linear regression correlation tests for CPUA of Round Goby and Percidae species in Big Otter Creek and the Ausable River, and the CPUA of the combined Percidae species in both tributaries. Sites where Round Goby CPUA = 0 were excluded

	Coefficients	Estimate	S.E	t	p	Adj. R^2^	Spearman
Big Otter Creek	Intercept	0.15	0.040	3.58	0.0011	− 0.032	0.016
	Johnny Darter	0.023	0.26	0.089	0.93		
	Intercept	0.13	0.04	3.4	0.0017	0.055	0.29
	Logperch	15	9.1	1.7	0.1		
	Intercept	0.17	0.049	3.6	0.0012	− 0.015	− 0.13
	Blackside Darter	− 1.1	1.4	− 0.73	0.47		
	Intercept	0.15	0.043	3.49	0.0015	− 0.032	0.0010
	All Percidae spp.	0.0013	0.23	0.006	0.99		
Ausable River	Intercept	0.13	0.062	2.0	0.06	− 0.075	0.04
	Johnny Darter	0.14	0.9	0.16	0.88		
	Intercept	0.11	0.059	1.8	0.09	− 0.042	0.18
	Logperch	3.1	4.7	0.66	0.52		
	Intercept	0.15	0.055	2.8	0.016	− 0.054	− 0.15
	Blackside Darter	− 3.8	7.1	− 0.53	0.6		
	Intercept	0.15	0.055	2.8	0.016	− 0.050	− 0.15
	Greenside Darter	− 0.27	0.48	− 0.56	0.58		
	Intercept	0.15	0.065	2.3	0.039	− 0.068	− 0.093
	All Percidae spp.	− 0.12	0.37	− 0.34	0.74		

### Aquatic habitat conditions

Stream physio-chemical conditions [conductivity (μS), dissolved oxygen (mg/L), pH, and turbidity (NTU)] varied along the length of the rivers (Table 3). Temperature did not differ meaningfully across site groupings in the Ausable River but was marginally cooler in the middle reach of Big Otter Creek (2.2–2.4 °C). The Ausable River became slower, deeper, and wider moving from the upstream to downstream sites. In Big Otter Creek, there were no meaningful differences in water velocities, depths, or widths. Organics, silts, and clays in the substrate of both rivers were consistent across site groupings, whereas % gravel increased from upstream to downstream in the Ausable River and decreased in the same direction in Big Otter Creek. Sands dominated the substrates of all sites in both rivers, with % sand tending to be higher in the lower site grouping of Big Otter Creek and consistent across site groupings in the Ausable River.

**Table 3:** Summary of mean site habitat conditions (± standard error) for site groupings in Big Otter Creek and the Ausable River

	Ausable River			Big Otter Creek		
	Site grouping			Site grouping		
	Lower	Middle	Upper	Lower	Middle	Upper
Stream order	6	6	4	5	5	5
Water temperature (°C)	22.7 (0.31)	21.6 (0.56)	22.7 (0.24)	22.2 ^**A**^ (0.63)	19.6^**B**^ (0.22)	22.4 ^**A**^ (0.61)
Conductivity (µS)	474^**A**^ (3.05)	472^**A**^ (8.03)	589^**B**^ (13.6)	564 ^**A**^ (2.51)	599^**B**^ (3.50)	544^**C**^ (7.46)
Dissolved oxygen (mg/L)	7.38^**A**^ (0.07)	7.36^**A**^ (0.20)	6.53^**B**^ (0.35)	9.10^**A,B**^ (0.31)	8.36^**A**^ (0.14)	10.2 ^**B**^ (0.41)
pH	8.28^**A**^ (0.02)	8.42^**B**^ (0.02)	8.29^**A**^ (0.04)	8.46^**A**^ (0.03)	8.37^**A**^ (0.02)	8.60^**B**^ (0.05)
Turbidity (NTU)	34.0^**A**^ (5.57)	31.9^**A**^ (7.04)	11.9^**B**^ (1.91)	26.8 (5.67)	13.6 (1.53)	9.35 (1.06)
Stream width (m)	17.3^**A**^ (2.81)	15.8^**A,B**^ (1.24)	10.4^**B**^ (0.89)	15.0 (0.56)	15.0 (1.25)	17.0 (4.98)
Stream depth (m)	1.1^**A**^ (0.13)	0.54^**B**^ (0.07)	0.46^**B**^ (0.04)	0.62 (0.04)	0.60 (0.06)	0.47 (0.12)
Stream velocity (m/s)	0.04^**A**^ (0.01)	0.11^**A,B**^ (0.03)	0.15^**B**^ (0.03)	0.28 (0.03)	0.25 (0.03)	0.24 (0.04)
% Organic	0.00 (0.00)	1.67 (0.94)	2.65 (1.49)	1.54 (1.54)	0.00 (0.00)	2.00 (2.00)
% Clay	10.6 (4.78)	0.00 (0.00)	4.71 (2.41)	0.00 (0.00)	0.00 (0.00)	0.00 (0.00)
% Silt	10.6 (3.92)	7.08 (2.57)	10.6 (2.64)	20.8 (2.39)	17.8 (2.78)	10.0 (5.48)
% Sand	38.8 (6.82)	27.1 (6.47)	34.4 (7.93)	68.5^**A**^ (6.19)	71.1^**A**^ (7.72)	34.0^**B**^ (9.27)
% Gravel	30.0^**A,B**^ (6.12)	41.7^**A**^ (6.92)	14.7^**B**^ (6.25)	5.38^**A**^ (3.32)	11.1^**A**^ (6.55)	42.0^**B**^ (12.0)
% Cobble	10.0 (3.65)	8.75 (3.15)	25.6 (8.26)	3.10 (3.08)	0.00 (0.00)	12.0 (7.35)
% Boulder	0.00 (0.00)	13.8 (9.28)	7.35 (4.31)	0.77 (0.77)	0.00 (0.00)	0.00 (0.00)
Dominant substrate (s)	Sand, Gravel	Sand, Gravel	Sand, Cobble	Sand	Sand	Sand, Gravel
Dominant floodplain use	Shrubs/Woodland	Shrubs/Woodland, Agricultural/Cropland	Shrubs/Woodland, Agricultural/Cropland	Agricultural/Cropland	Agricultural/Cropland	Shrubs/Woodland

Bold superscripts denote where mean habitat conditions differed significantly (*p* < 0.05) among site groupings within each tributary

### Round Goby influence on fish community metrics

**Fig. 4:**
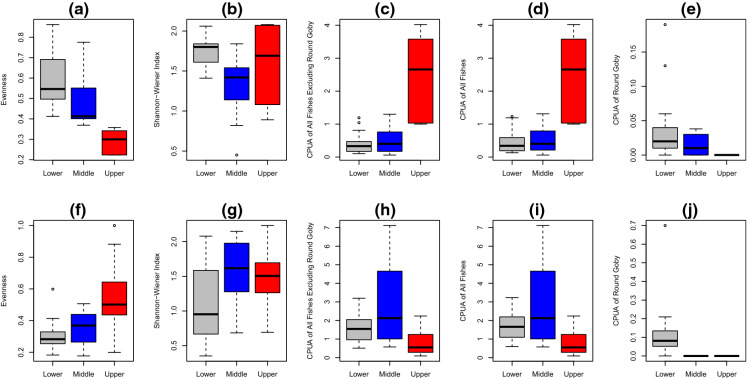
Boxplots comparing diversity metrics between site groupings in Big Otter Creek (top, **a–e**; lower sites: 13.1–14.1 km from river mouth, n = 13; middle sites: 35.8–49.9 km from river mouth, n = 9; upper sites: 71.2–83.6 km from river mouth, n = 5), and the Ausable River (bottom, **f–j**; lower sites: 11.2–17.7 km from river mouth, n = 16; middle sites: 32.1–75.6 km from river mouth, n = 12 sites; upper sites: 91.6–126.4 km from river mouth, n = 17). Diversity metrics include: Evenness (**a, f**), Shannon–Wiener Index (**b, g**), CPUA of all sampled fishes excluding Round Goby (**c, h**), CPUA of all sampled fishes including Round Goby (**d, i**), and CPUA of Round Goby. Boxplots represent the median, minimum, maximum, and first and third quartiles of sites in each site grouping

In the Ausable River, evenness was lowest in the lower zone sites, and higher in the upper zone sites, but followed the reverse pattern in Big Otter Creek with the highest evenness in the lower zone sites and lowest in the upper zone sites (Fig. 4a, f). In the Ausable River, the Shannon–Wiener index was highest in the middle zone sites, whereas in Big Otter Creek the index was highest in the lower and upper zone (Fig. 4b, g). The CPUA of all fishes (including and excluding Round Goby) was highest in sites in the middle zone of the Ausable River, but highest in the upper zone sites of Big Otter Creek (Fig. 4d, i). As seen in Figs. 1, 2, 3, 4e, and 4j, Round Goby CPUA was highest in the lower sites and lowest in the middle and upper zones of both tributaries.

**Fig. 5:**
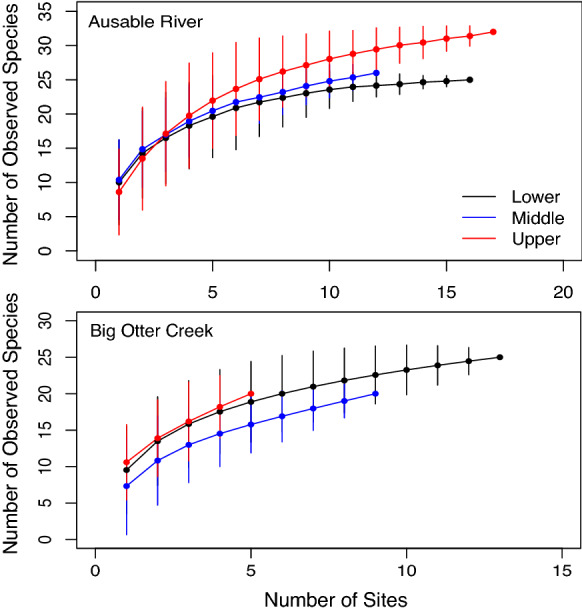
Species accumulation curves for Big Otter Creek (lower sites: 13.1–14.1 km from river mouth, n = 13; middle sites: 35.8–49.9 km from river mouth, n = 9; upper sites: 71.2–83.6 km from river mouth, n = 5) and the Ausable River (lower sites: 11.2–17.7 km from river mouth, n = 16; middle sites: 32.1–75.6 km from river mouth, n = 12 sites; upper sites: 91.6–126.4 km from river mouth, n = 17). Error bars are ± one standard deviation

The species accumulation curve for the upper zone of the Ausable River displayed a higher trajectory and contained higher species richness than the lower and middle zones (Fig. 5). Both the lower and middle zones of the Ausable River followed a similar trajectory with similar observed species richness. The species accumulation curve for the upper and lower zones of Big Otter Creek were similar and displayed slightly higher observed species richness than the middle zone, but overall, site groupings appeared to have a similar number of observed species (Fig. 5). In the Ausable River and Big Otter Creek, the middle zone had higher estimated species richness than the lower and upper zones (Table 4). The middle zone contained the second lowest and lowest total CPUA of Round Goby in the Ausable River and Big Otter Creek, respectively. The lowest estimated richness occurred in the lower zone of both tributaries where total Round Goby CPUA was highest. Fish communities in Big Otter Creek showed low similarity between site groupings (lower and middle *C*_*H*_ = 0.31, lower and upper *C*_*H*_ = 0.26, middle and upper *C*_*H*_ = 0.05). In the Ausable River, site groupings showed high similarity between the lower and middle (*C*_*H*_ = 0.88) and the lower and upper (*C*_*H*_ = 0.97) zones, but moderate similarity between the middle and upper (*C*_*H*_ = 0.38) zones. There was no evidence for spatial autocorrelation between sites at the analyzed spatial scale in Big Otter Creek: Round Goby (*I* = − 0.30, standard deviation = 0.09, *p* = 0.71), Percidae species (*I* = − 0.13, standard deviation = 0.11, *p* = 0.06) or the Ausable River: Round Goby (*I* = − 0.19, standard deviation = 0.03, *p* = 0.62), Percidae species (*I* = − 0.23, standard deviation = 0.05, *p* = 0.57).

**Table 4:** Summary of Chao1 local richness estimates from each site grouping and the mean CPUA of Round Goby in sites within the site groupings in each tributary

Tributary	Site grouping	Number of species	Number of sites	Chao1 (± S.E.)	Mean Round Goby CPUA
Big Otter Creek	Lower	25	13	32.5 (7.68)	0.04
	Middle	20	9	56 (43.7)	0.01
	Upper	20	5	36.2 (16.2)	0.0
Ausable River	Lower	25	16	26.4 (2.17)	0.13
	Middle	26	12	48.5 (28.5)	0.0
	Upper	32	17	39.5 (7.07)	0.0

In the Ausable River, Round Goby represented 7.36% of the CPUA in the lower grouping but was not captured elsewhere (Table 5). The dominance in the lower reaches of Leuciscidae remained stable (79.4–72.4%) as sampling moved to the upper reaches. Percidae (7.3–15.1%) and Centrarchidae (0.2–9.5%) both increased in importance when moving from the lower to the upper reaches. Similar declines in Round Goby were observed in Big Otter Creek, with percentage importance in the catch declining from 8.6 to 0% as sampling moved upstream. Leuciscidae showed no noticeable trend in relative abundance, remaining between 60.2 and 69.6% of the total catch along the length of the creek. Catostomidae, which had an only minor but stable presence in the Ausable River, were a significant portion of the catch at all sites (9–19.3%). Percidae, which peaked in percentage importance in the middle reaches (26.4%) showed no discernable trend and was a significant proportion of the catch in both the lower (11.8%) and upper (13.3%) reaches of Big Otter Creek. The most notable trend was the increase in Leuciscidae from 5.3% in the lower reaches to 11.6% in the upper reaches, a trend that was the direct opposite of that observed in the Ausable.

**Table 5:** Summary of % CPUA (fish × (m^2^)^−1^) from site groupings in the Ausable River and Big Otter Creek

			Ausable River			Big Otter Creek		
Family	Species		Site Grouping			Site Grouping		
	Common Name	Latin Name	Lower	Middle	Upper	Lower	Middle	Upper
Atherinopsidae								
	Brook Silverside	*Labidesthes sicculus*	1.97	0	0	0	0	0
Catostomidae								
		*Catostomidae spp.*	0	0	0	0.64	0.91	1.30
	White Sucker	*Catostomus commersonii*	0.29	0.18	0.28	17.5	7.83	12.1
	Northern Hogsucker	*Hypentelium nigricans*	0.04	0.45	1.85	0.79	0.21	2.49
	Golden Redhorse	*Moxostoma erythrurum*	0.98	0.03	0.28	0	0	0.08
	Shorthead redhorse	*Moxostoma macrolepidotum*	0	0.15	0	0	0	0.10
		*Moxostoma spp.*	0.18	0	0	0.16	0	0
	Greater Redhorse	*Moxostoma valenciennesi*	0.46	0	0	0.16	0	0
Centrarchidae								
	Rock Bass	*Ambloplites rupestris*	0	0.23	4.94	0	0	0
	Pumpkinseed Sunfish	*Lepomis gibbosus*	0	0	0.52	0.16	0	0
	Bluegill Sunfish	*Lepomis macrochirus*	0	0.07	0	0	0	0
	Northern Sunfish	*Lepomis peltastes*	0	0	0.57	0	0	0
		*Lepomis spp.*	0	0	0.09	0	0	0
	Smallmouth Bass	*Micropterus dolomieu*	0.17	0.91	3.27	0	0.21	0
	Black Crappie	*Pomoxis nigromaculatus*	0	0	0.09	0	0	0
Clupeidae								
	American Gizzard Shad	*Dorosoma cepedianum*	0.20	0	0	0	0	0
Esocidae								
	Northern Pike	*Esox lucius*	0	0	0.28	0	0	0
Gasterosteidae								
	Brook Stickleback	*Culaea inconstans*	0	0	0	0	0	1.06
Gobiidae								
	Round Goby	*Neogobius melanostomus*	7.36	0	0	8.59	2.43	0
Ictaluridae								
	Black Bullhead	*Ameiurus melas*	0	0	0.07	0	0	0
	Yellow Bullhead	*Ameiurus natalis*	0	0	0.23	0	0	0
	Channel Catfish	*Ictalurus punctatus*	1.25	0.07	0	0	0	0
	Stonecat	*Noturus flavus*	0.14	0.20	0.08	0.16	0	0
	Tadpole Madtom	*Noturus gyrinus*	0	0	0	0	0.41	0
Leuciscidae								
	Central Stoneroller	*Campostoma anomalum*	0	0.16	0.97	0	0	0.16
	Northern Redbelly Dace	*Chrosomus eos*	0	0	0	0	0	1.30
	Emerald Shiner	*Notropis atherinoides*	2.57	1.19	0.07	0	0	0
	Ghost Shiner	*Notropis buchanani*	65.7	1.99	0	0	0	0
	Spottail Shiner	*Notropis hudsonius*	0	0	0	0.16	0	0
	Rosyface Shiner	*Notropis rubellus*	0	28.0	17.3	1.75	1.03	0.49
		*Notropis spp.*	0	0	0.36	0	0	0
	Mimic Shiner	*Notropis volucellus*	6.04	25.0	1.78	2.86	0.82	0
	Eastern Blacknose Dace	*Rhinichthys atratulus*	0	0	0.07	0.48	0.21	5.75
	Longnose Dace	*Rhinichthys cataractae*	0	0	0	0	4.09	3.92
	Spotfin Shiner	*Cyprinella spiloptera*	0.91	5.75	8.10	8.43	1.85	0
		*Cyprinidae spp.*	1.76	0.25	0.07	0.16	0	0.08
	Striped Shiner	*Luxilus chrysocephalus*	0.06	1.54	5.78	0	0	0
	Common Shiner	*Luxilus cornutus*	0.22	2.23	13.1	23.1	46.0	42.8
		*Luxilus spp.*	0	0	6.46	9.54	0.41	2.28
	Northern Pearl Dace	*Margariscus nachtriebi*	0	0	0	0	0	0.08
	Hornyhead Chub	*Nocomis biguttatus*	0	0.08	8.39	0	0	0
	River Chub	*Nocomis micropogon*	0.24	1.47	1.08	8.90	1.64	0
		*Nocomis spp.*	0	0	0	0.48	0	0
	Golden Shiner	*Notemigonus crysoleucas*	0	0	0	0.32	0	0
	Bluntnose Minnow	*Pimephales notatus*	1.85	12.7	8.84	0.95	1.03	1.63
	Fathead Minnow	*Pimephales promelas*	0	0	0	0	0.21	0.16
	Creek Chub	*Semotilus atromaculatus*	0	0.07	0	3.02	4.32	10.9
Percidae								
	Greenside Darter	*Etheostoma blennioides*	3.80	9.83	3.88	0	0	0
	Rainbow Darter	*Etheostoma caeruleum*	0	0	1.31	0	0	0
	Johnny Darter	*Etheostoma nigrum*	2.74	4.41	7.50	3.34	22.0	12.3
	Yellow Perch	*Perca flavescens*	0	0	0	1.59	0.21	0
	Logperch	*Percina caprodes*	0.50	0.23	0.61	0.16	0	0
	Blackside Darter	*Percina maculata*	0.26	2.84	1.80	6.68	4.21	0.98
Percopsidae								
	Trout-Perch	*Percopsis omiscomaycus*	0.26	0	0	0	0	0

## Discussion

We found evidence to suggest that Round Goby has negatively affected the relative abundance of several darter species and the overall fish community structure in invaded riverine ecosystems of the Great Lakes. However, overwhelming evidence of negative associations with darter species was not found. Round Goby relative abundance was highest proximate to the Great Lakes but sharply decreased thereafter, with upstream reaches in both tributaries having higher relative abundances of darter species: Greenside Darter, Blackside Darter, and Johnny Darter in the Ausable River, and Johnny Darter in Big Otter Creek. Significant negative correlations of CPUA between all darter species and Round Goby were not observed, with co-occurrence patterns indicating negative associations only for a subset of species (Ausable River: Rainbow Darter, Big Otter Creek: Johnny Darter and Percidae overall). Overall diversity, species richness, evenness, relative abundance, and species accumulation curves for fishes in the studied tributaries demonstrated variation across site groupings despite the similarity of stream habitat conditions, particularly substrate, between site groupings. The observed patterns of effect also differed between the rivers.

The general pattern of low darter relative abundance in sites with high Round Goby relative abundance suggests that Round Goby may have reduced the populations of several darter species, as has been noted for other tributaries of the Great Lakes (Krakowiak and Pennuto 2008; Raab et al. 2018). Alternatively, Round Goby may have triggered a habitat redistribution effect for darters (e.g. Greenside, Blackside, and Johnny Darters within the studied tributaries, as has been shown to occur elsewhere as a consequence of invasive fishes (Habit et al. 2010). For example, the seasonal dispersal of non-native Brown Trout (*Salmo trutta*) from Czech reservoirs alters the spatial distribution of native fishes in tributary streams (Pfauserová et al. 2021). Furthermore, experimental studies have demonstrated the redistributive effect, having shown that benthic fish species can display significant shifts in riverine habitat use when co-occurring with invasive gobiids, moving from preferred shelter habitat to less preferred and riskier habitats subject to predation (Van Kessel et al. 2011). Thus, the rise in the relative abundance of Greenside, Blackside, and Johnny Darters in the Ausable River and Johnny Darter in Big Otter Creek may have been an artefact of redistribution caused by avoidance behaviour (Ayala et al. 2007).

Invasive species initially negatively affect the more abundant native species before affecting rarer native species (Powell et al. 2013). Based on the correlated reduction in abundant darter species along the tributary gradients, both tributaries may still be experiencing the early-stage effects of a Round Goby invasion given that Round Goby CPUA values in this study were comparable to those found shortly after introduction in the Flint River between 1998 and 2002 (0.29–0.78 individuals/m^2^, Carman et al. 2006; Jude et al. 2018) and in Lake Michigan tributaries between 2007 and 2010 (0.07–0.36 individuals/m^2^, Kornis et al. 2013). Similar to other invasive species, Round Goby has been noted to rapidly attain high densities during the early stages of invasion before stabilizing or declining in numbers as the invasion progresses (Young et al. 2010; Kornis et al. 2012, 2014; Burkett and Jude 2015). For example, in Hamilton Harbour, it was proposed that declines in Round Goby abundance resulted from the exceedance of their ecological carrying capacity (Young et al. 2010).

Round Goby populations at higher densities have also been stabilized through predatory control. In Lake Erie, after dramatic population increases, Round Goby experienced population declines following the development of predatory control by Burbot (*Lota lota*) (Madenjian et al. 2011). In the St. Clair River, the diets of large Rock Bass (*Ambloplites rupestris*) and large Smallmouth Bass (*Micropterus dolomieu)* have been shown to consist of 56–67% and 100% Round Goby, respectively (Burkett and Jude 2015), with similar results reported from the River Dyje in the Czech Republic (Mikl et al. 2017). Predation control, however, appears unlikely to be occurring in either of the streams studied here, with cumulative catches of predatory or partially predatory fishes (e.g. Rock Bass, Smallmouth Bass, Northern Pike, Yellow Perch) in both rivers never exceeding 8.7% of the total CPUA and most reaches never exceeding 2% of the total CPUA.

The low relative abundance of darter species in areas of high Round Goby relative abundance observed along the tributary gradients may also be driven by competitive interactions. For example, one recent study assessing the trophic impacts of Round Goby on native benthic fishes in the Sydenham River (also a tributary of the Great Lakes) found diet overlap between Round Goby and several darter species including: Greenside Darter, Blackside Darter, Johnny Darter, and the Threatened Eastern Sand Darter (Firth et al. 2020). In addition to competition for similar food resources, Round Goby has been shown to outcompete other species for habitat due to their aggressive behaviour and ability to achieve greater body sizes than other similar species (Charlebois et al. 2001; Balshine et al. 2005) and, as has been noted in laboratory experiments, has the capacity to displace native benthic fishes from more sheltered, desirable habitats (Van Kessel et al. 2011). Thus, interspecific competition for habitat and/or dietary resources may account for the low relative abundance of darter species observed in the downstream reaches with high Round Goby relative abundance and the moderately negative co-occurrences between Round Goby and several darter species. Continued competition and/or displacement of native darter species by Round Goby as the invasion front advances would contribute to further declines in riverine darter populations (Poos et al. 2010; Firth et al. 2020).

Increases in Round Goby populations have led to declines to native fishes in other freshwater environments, most notably in several major European rivers (Danube River and River Rhine) and their tributaries. The continued dispersal and increase in abundance of Round Goby is expected to further increase competition with native fishes in those systems (Borcherding et al. 2011; Cerwenka et al. 2018; Dashinov and Uzunova 2020). In North America, Round Goby in the Great Lakes has also caused declines to numerous native benthic fishes (e.g. *Etheostoma spp*., *Percina spp*., and Cottus *spp.*) via competition for food, habitat, and spawning sites (French and Jude 2001; Lauer et al. 2004; Reid and Mandrak 2008; Abbett et al. 2013). Accordingly, Round Goby is renowned for its ability to outcompete other fishes through both interference and exploitative competition. Laboratory studies have provided evidence that Round Goby can outcompete Spoonhead sculpin (*Cottus ricei),* Slimy sculpin (*Cottus cognatus*), and Logperch for optimal habitat and food resources (Balshine et al. 2005; Bergstrom and Mensinger 2009). However, without direct evidence of overlap in resource use between Round Goby and Percidae species in the Ausable River and Big Otter Creek (i.e., through gut content analysis or stable isotope analysis), uncertainty exists as to the mechanisms through which Round Goby may be contributing to the negative co-occurrences with some darters and the observed low darter abundances in downstream reaches.

While several studies have investigated the impacts of Round Goby invasion in tributaries flowing directly into the Great Lakes, their scope has been confined to assessing impacts at limited spatial scales and in areas proximate to the lakes. For example, Krakowiak and Pennuto (2008) found that darter species (Johnny Darter and Rainbow Darter) historically present in streams before Round Goby establishment were absent after Round Goby establishment at sites located 2–3 km from Lake Erie where Round Goby were already known to occur in high nearshore densities. Similarly, in both the Ausable River and Big Otter Creek, abundances of Johnny Darter increased upstream of sites proximate to the lakes, with the study of resident fish communities at greater longitudinal scales demonstrating a Round Goby density gradient effect on native darter abundances. In addition, effects found here may be related to the longer post-invasion intervals (11–16 years) considered in our study. Studies that have investigated Round Goby impacts in tributaries shortly after establishment may not accurately reflect impacts to native fish communities over prolonged time periods. Long-term studies across large spatial scales in European rivers have demonstrated considerable variability in the observed impacts of Round Goby on native fish communities. Round Goby became the most abundant fish species in a 248 km stretch of the upper Danube River ten years after its introduction (Cerwenka et al. 2018). However, the effects from Round Goby populations in the Danube River differ across spatial gradients depending on invasion stage. Individuals at the invasion fronts have larger bodies and greater body condition than those in already established areas due to weaker intraspecific competition and greater food availability at the invasion fronts (Brandner et al. 2018). Although invasion impacts to aquatic ecosystems may be apparent after short time periods (Janssen and Jude 2001; Lauer et al. 2004; Britton et al. 2007; Connelly et al. 2007), it is necessary to assess impacts at longer post-invasion intervals to ultimately understand both how the invader will influence the ecosystem and how impacts are related to the invasion stage (Downing et al. 2013; Pelicice et al. 2014; Havel et al. 2015).

Fish community diversity metrics and abundances of Round Goby and Percidae species will have been influenced by habitat conditions, which are integral in shaping fish communities within streams (Moyle and Light 1996). Abiotic conditions (e.g. watershed area and temperature) were best at predicting Round Goby abundance in Lake Michigan tributaries (Kornis et al. 2013) and thus variation in Round Goby abundance may have been reflective of differences in habitat suitability along the lengths of the studied rivers. Round Goby has shown preference for a wide variety of habitat conditions in tributaries, occupying sites dominated by cobble, gravel, sand, or silt substrate (Pennuto et al. 2018; Reid 2019), slow to moderate water velocity (< 0.4 m/s; Raab et al. 2018; Reid 2019), and a wide range of water depths (0.2–2.2 m; Raab et al. 2018; Reid 2019). The preference for slow water velocity may explain why Round Goby density was high in the lower reach of the Ausable River where mean water velocity was lowest (0.04 m/s). Slow water velocity may also account for the anomaly in the middle grouping site 58 km upstream in Big Otter Creek where there is a higher relative abundance of Round Goby (CPUA = 0.32) than nearby sites. The notably slower water velocity (0.07 m/s) in that site than typically seen in the other middle grouping sites (mean = 0.25 m/s) of Big Otter Creek may have provided more favourable conditions for Round Goby. Water velocity and other habitat features likely play a role in shaping Round Goby populations in streams, particularly as Round Goby has shown preferences for volitional movement under low flow conditions (Tierney et al. 2011).

Several populations of Round Goby in the Great Lakes (Lake Erie, Lake Ontario) undergo seasonal migration into tributaries in the summer months followed by migration back to the lake in winter months (Blair et al. 2019). Tributary streams connected to large, invaded waterbodies (i.e. the Great Lakes) could be used primarily for reproduction and recruitment and Round Goby may not actually reside in the tributaries year-round (Pennuto et al. 2010, 2021; Blair et al. 2019). The high relative abundance of Round Goby observed in tributary sites adjacent to the Great Lakes is consistent with a pattern of seasonal migration into the Ausable River and Big Otter Creek and may explain the predominantly lower reach effects observed here. Indeed, seasonal pulse effects of non-native species on native species have been observed elsewhere where migration from lake habitats to tributaries is possible (Pfauserová et al. 2021). Nevertheless, Round Goby detections in Big Otter Creek 62 km upstream are suggestive of permanent, year-round populations. If populations of Round Goby permanently reside in lower reaches of the tributaries and dispersal and expansion continue to upstream reaches (Kornis et al. 2012, 2013), then native fishes will likely experience more sustained and consistent competition with Round Goby. Based on effects observed here, increasing Round Goby densities along greater expanses of the studied tributaries have the potential to lead to further darter and native benthic fish population declines and associated reductions in fish community diversity.

The assessment of the longitudinal changes in the relative abundance of Round Goby and native fish species and the associated impacts of Round Goby on fish community metrics in our tributaries provides evidence of the negative effects of Round Goby invasions on the relative abundance of key darter species. However, significant negative correlations between the CPUA of darter species and Round Goby were not observed, with co-occurrence patterns indicating moderately negative associations only for a subset of species (Ausable River: Rainbow Darter, Big Otter Creek: Johnny Darter and Percidae overall). Findings corroborate earlier studies but increase understanding of the longitudinal aspects of invasion impacts. As sampling did not directly address issues of inter-annual variability, temporal trends involving the relative abundances of fishes and fish community diversity metrics remain unknown in these particular tributaries. Accordingly, further sampling in Great Lakes tributaries is suggested as necessary for better evaluating Round Goby impacts across time and space and to gain greater understanding of how native fish communities will be ultimately influenced by this invader.

## Data Availability

All data generated and analyzed during this study are included in Barnucz et al. (2020).
